# THE FLYNN EFFECT IN SHORT-TERM COGNITIVE DECLINES OF AMERICANS AGED 65 YEARS AND OLDER: SMARTER AND MAYBE SLOWER

**DOI:** 10.1093/geroni/igac059.916

**Published:** 2022-12-20

**Authors:** Yun Zhang, Joeseph Rodgers, Patrick O'Keefe, Graciela Muniz Terrera, Stacey Voll, Frank D Mann, Scott Hofer, Sean Clousten

**Affiliations:** Stony Brook University, Stony Brook, New York, United States; Vanderbilt University, Nashville, Tennessee, United States; Oregon Health and Science University, Portland, Oregon, United States; Ohio University, Athens, Ohio, United States; University of Victoria, Victoria, British Columbia, Canada; Stony Brook University, New York, New York, United States; University of Victoria, Victoria, British Columbia, Canada; Stony Brook University, Stony Brook, New York, United States

## Abstract

To contribute to our understanding of cohort differences and the Flynn Effect in cognitive declines, this study aims to: 1) describe and compare cognitive decline trends of two nationally representative American older cohorts; 2) investigate significant determinants of cognitive declines and the cohort differences. The analysis used data from the National Health and Aging Trends Study (NHATS, 2011-2019), including one nationally representative cohort of older Americans in 2011 and another from 2015. We used mixed-effect models adjusted for cohort, wave, baseline age, sex, education, race, familiarity, and follow-up years, as well as survey designs, to describe and compare the intercepts and slopes in cognitive functions of the two NHATS cohorts. We included Cohort 1 (N=7,325) respondents (2011-2015), and Cohort 2 (N=7,330) respondents (2015-2019). Compared to Cohort 1, Cohort 2 has a significantly higher intercept and a slower decline for episodic memory, and a significantly higher intercept but a significantly faster decline for global cognition, orientation function, and executive function. Consistently, older age, poorer educational attainment, and minority races/ethnicity are associated with worse cognitive performances. Our results provide a comprehensive image of cohort declines for Americans aged 65 years and older. Our findings are consistent with the Flynn Effect in that the general levels of cognition of later cohorts improved. Furthermore, we found support for the Flynn Effect in a short term. We also found significant effects of older age, poorer educational attainment, and minority race/ethnicity on cognitive function.

